# Basal tolerance to heat and cold exposure of the spotted wing drosophila, *Drosophila suzukii*

**DOI:** 10.7717/peerj.3112

**Published:** 2017-03-23

**Authors:** Thomas Enriquez, Hervé Colinet

**Affiliations:** Université de Rennes I, UMR CNRS 6553 ECOBIO, Rennes, France

**Keywords:** Spotted wing Drosophila, Cold tolerance, Heat tolerance, Realtive humidity, Thermal tolerance landscape

## Abstract

The spotted wing Drosophila, *Drosophila suzukii*, is a new pest in Europe and America which causes severe damages, mostly to stone fruit crops. Temperature and humidity are among the most important abiotic factors governing insect development and fitness. In many situations, temperature can become stressful thus compromising survival. The ability to cope with thermal stress depends on basal level of thermal tolerance. Basic knowledge on temperature-dependent mortality of *D. suzukii* is essential to facilitate management of this pest. The objective of the present study was to investigate *D. suzukii* basal cold and heat tolerance. Adults and pupae were subjected to six low temperatures (−5–7.5 °C) and seven high temperatures (30–37 °C) for various durations, and survival-time-temperature relationships were investigated. Data showed that males were globally more cold tolerant than females. At temperature above 5 °C, adult cold mortality became minor even after prolonged exposures (e.g., only 20% mortality after one month at 7.5 °C). Heat tolerance of males was lower than that of females at the highest tested temperatures (34, 35 and 37 °C). Pupae appeared much less cold tolerant than adults at all temperatures (e.g., Lt_50_ at 5° C: 4–5 d for adults *vs.* 21 h for pupae). Pupae were more heat tolerant than adults at the most extreme high temperatures (e.g., Lt_50_ at 37 °C: 30 min for adults *vs.* 4 h for pupae). The pupal thermal tolerance was further investigated under low *vs.* high humidity. Low relative humidity did not affect pupal cold survival, but it reduced survival under heat stress. Overall, this study shows that survival of *D. suzukii* under heat and cold conditions can vary with stress intensity, duration, humidity, sex and stage, and the methodological approach used here, which was based on thermal tolerance landscapes, provides a comprehensive description of *D. suzukii*thermal tolerance and limits.

## Introduction

Temperature and water availability are among the most important factors influencing animal distribution, reproduction, and fitness (Chown & Nicolson, 2004; Angilletta Jr , 2009) and has therefore a great influence on the invasive success of alien species (Bellard et al., 2013). The spotted wing Drosophila (SWD), *Drosophila suzukii* (Matsumara), is a new insect pest in Western Europe and North America that causes severe damage to a large range of fruit plants, and especially stone fruit crops (Walsh et al., 2011; Asplen et al., 2015). This invasive species is native to Southeast Asia and has been introduced in Spain, Italy, and North America in 2008 (Hauser, Gaimari & Damus, 2009; Raspi et al., 2011; Calabria et al., 2012). It is now widely distributed in West Europe (Cini, Ioratti & Anforta, 2012) and both in United States and in Southern Canada (Hauser, 2011). While most *Drosophila* species oviposit in rotting fruits, SWD females prefer to oviposit in ripe fruits (Kanzawa, 1939; Mitsui, Takahashi & Kimura, 2006). A sclerotized ovipositor allows flies to pierce through skin fruit (Hauser, Gaimari & Damus, 2009), and lay their eggs into a very large host range (Cini, Ioratti & Anforta, 2012; Poyet et al., 2015). The damages that larvae cause to fruits can have huge economic impact (Goodhue et al., 2011). Insect resistance to chemicals, frequent applications of insecticides owing to SWD short generation time and concerns about public health are considerable issues that have turned research towards non-chemical, environmentally friendly approaches. Sterile Insect Technique (SIT) or Incompatible Insect Technique (IIT) are innovative methods that offer sustainable opportunities to regulate pest populations (Zabalou et al., 2004; Dyck, Hendrichs & Robinson, 2005). Efforts are currently underway to develop such techniques in SWD (H Colinet, pers. comm., 2017). These technologies require release of a huge number of competitive insects produced at industrial scale. A successful application of SIT and/or IIT thus requires basic knowledge on thermal biology of SWD to develop cold storage methods and adapt prior-release mass-rearing protocols to the temperature within the release site(s) (*e.i.* in greenhouse).

It is assumed that the success of SWD invasion is partly due to a series of adaptations to temperate climates (Rota-Stabelli, Blaxter & Anfora, 2013). For instance, this species is freeze-intolerant and chill-susceptible (Kimura, 2004; Dalton et al., 2011; Jakobs, Gariepy & Sinclair, 2015; Ryan et al., 2016; Plantamp et al., 2016) but has a large thermal tolerance plasticity which likely favors its overwintering (Jakobs, Gariepy & Sinclair, 2015). Another hypothesis for explaining its overwintering success in cold regions is that adults may take refuge into human-made structures or migrate to suitable microclimates during cold periods (Kanzawa, 1939; Kimura, 2004; Cini, Ioratti & Anforta, 2012; Rota-Stabelli, Blaxter & Anfora, 2013; Zerulla et al., 2015). SWD overwinters as adult dark winter morph (Kanzawa, 1936; Stephens et al., 2015; Shearer et al., 2016; Toxopeus et al., 2016; Wallingford & Loeb, 2016). This morph is characterized by arrested reproduction and increased cold tolerance (Stephens et al., 2015; Toxopeus et al., 2016; Shearer et al., 2016; Wallingford & Loeb, 2016), but it is not yet clear whether this morph entails a true reproductive diapause or not (Toxopeus et al., 2016; Wallingford & Loeb, 2016; Zhai et al., 2016). Most recent studies on SWD cold tolerance were designed to understand overwintering strategy in newly infested cold regions, in order to better predict invasion potential or winter survival probability (*e.i.* Dalton et al., 2011; Stephens et al., 2015; Zerulla et al., 2015; Shearer et al., 2016; Wallingford & Loeb, 2016). In most of these studies, cold survival was assessed by subjecting insects either to a single low temperature with different durations (*e.i.* Jakobs, Gariepy & Sinclair, 2015; Toxopeus et al., 2016), or to a series of low temperatures but with a single duration of exposure (*e.i.* Kimura, 2004; Ryan et al., 2016; Wallingford & Loeb, 2016). From a conceptual perspective, this can be questionable as the impact of any stress depends on its intensity and duration. Hence, investigating a single parameter may not be sufficient to describe a response that is embedded in two dimensions (Rezende, Castañeda & Santos, 2014). To fully appreciate the innate capacity of a species to cope with cold and heat stress, an approach based on tolerance landscape was suggested by Rezende, Castañeda & Santos (2014). The thermal tolerance landscape (TTL), describes the probability to survive thermal stress as a function of both the intensity and the duration. In the present study, this approach was adopted to describe basal heat and cold tolerance of SWD at adult and pupal stage. Previous works have reported basic information on heat tolerance of SWD in native SWD populations (Kanzawa, 1939; Kimura, 2004). Kanzawa (1939) found a decrease of SWD motor activity when exposed to 30 °C, and Kimura (2004) exposed flies at different temperatures for a single duration of 24 h and estimated the 50% lethal temperature to be at 32 °C. Since invasion process may trigger adaptation to novel climatic areas, data are highly needed on invasive populations submitted to a range of thermal stress intensities and durations. In *Drosophila melanogaster*, the humidity during thermal stress modifies survival probability and the response can be temperature-dependent (Bubliy et al., 2012; Kobey & Montooth, 2013). Combining high temperature with low humidity provides more stressful conditions to *Drosophila* flies than high temperature with high humidity (Bubliy et al., 2012). Furthermore, increasing humidity during cold exposure increased survival at 6 °C, but not at −4 °C in *D. melanogaster* (Kobey & Montooth, 2013). This underlines that interaction among abiotic factors may be complex. In the present study, we also investigated the effect of different relative humidity levels (low *vs*. high) on cold and heat tolerance of SWD pupae. The general assumptions of this study were: (1) SWD survival would be a function of both temperature stress intensity and time exposure. (2) Because thermal performance curves are nonlinear and asymmetric (Colinet et al., 2015a), we predicted uneven effects of increasing intensity of cold and heat stress. More specifically, we predicted that survival would decrease rather progressively with cold stress intensity, and more abruptly with heat stress intensity. (3) We also expected differential responses between sexes and stages. Based on previous data from *D. melanogaster* (Jensen, Overgaard & Sørensen, 2007), we predicted that pupae would be less thermotolerant than adults. (4) Finally, we predicted that desiccating condition during thermal stress would further reduce survival probability compared to thermal stress under high humidity.

## Materials and methods

### Flies origin and rearing

The SWD strain is initially based on a mixed population from infested fruits collected from different locations in the south part of the Sugana Valley (Trentino, Italia), and brought to the Vigalzano station of the Edmund Mach foundation (46°04′25.74″N 11°13′52.45″E) in 2011. This strain was then exported in our laboratory (Rennes/France) in early 2016 where it has been continuously mass-reared in high numbers. For experimentations, SWD was reared in glass bottle (100 mL) and supplied with standard diet (for 1 l: agar: 15 g, sucrose: 50 g, brewer yeast: 40 g, cornmeal: 40 g, kalmus: 8 g, Nipagin: 8 mL). At least 12 bottles (each containing 100–300 flies) were used to maintain the strain, and flies from different bottles were crossed every generation to limit inbreeding. Bottles were kept in incubators (Model MIR-154-PE; PANASONIC Healthcare Co., Ltd. Gunma, Japan) at 25 °C, 65–70% RH, 12L : 12D. Adults and pupae randomly taken from the rearing stock were used in experiments. In the present study, we focused on adults and pupae as these two stages can be isolated from the food and are thus more convenient for cold storage than larvae. All tested adults were between 5- and 7-days-old to avoid age-related differences in stress tolerance (Colinet et al., 2015b). Males were separated from females visually (with an aspirator) without CO_2_ to avoid stress due to anesthesia (Colinet & Renault, 2012). For pupae, individuals that had pupated for a maximum of 48 h were used (i.e., corresponding to eight days after egg laying at 25 °C).

### Thermal tolerance assays

Flies and pupae were subjected to six low constant temperatures (−5, −2.5, 0, 2.5, 5 and 7.5 °C) and seven high constant temperatures (30, 31, 32, 33, 34, 35 and 37 °C) for various durations. At least seven durations were used for each temperature and these are provided in Tables S1–S5 for each experiment. Exposure durations were pre-determined with preliminary assays, in order to obtain for each temperature a range of survival spanning from 0% for the shortest exposure to 100% for the longest exposure. At the most stressful temperatures (at heat and cold), additional time points were considered because mortality occurred very quickly (within less than 2 h) (see Tables S1–S5). For each sampling duration, three replicates of 10 flies or 10 pupae, randomly taken from the rearing stock, were used. Flies and pupae were exposed to the different thermal conditions either using food vials placed in incubators (Model MIR-154-PE; PANASONIC Healthcare Co., Ltd. Gunma, Japan) for the longer assays (2.5, 5, 7.5, 30, 31, 32 °C) or using glass vials immersed in a glycol solution cooled by a cryostat (Cryostat Lauda ECO RE 630) for the shorter assays (−5, −2,5, 0, 33, 34, 35, 36, 37 °C). Males and females were exposed in separated vials. Temperature was checked during all assays using thermocouple K (measuring accuracy: ±0.5 °C) connected Testo thermometers (Model 175 T3; TESTO Limited, Hampshire, England) placed into the same experimental vial without flies. After stress exposure, SWD adults were allowed to recover in 40 mL food vials under standard rearing conditions (25 °C, 65–70% RH, 12L : 12D). Adult survival was assessed by counting the proportion of dead and living individuals 24 h post exposure. For pupae, the results were expressed as a percentage of eclosion, considered here as a proxy of survival. Flies were considered as alive when the adult totally emerged from the puparium. Because isolation and manipulation of pupae in the preparation of thermal assays might cause some damage to the puparium, five sets of 20 untreated pupae were kept at 25 °C to estimate possible mortality due to manipulation.

### Thermal stress under high and low relative humidity

In this experiment, we used only pupae to assess the impact of humidity (low *vs*. high) during thermal stress. Indeed, there was a technical limitation with the application of low RH in adults, as it was impossible to generate low RH (5–10%) with presence of food within the vials. Pupae were exposed to four different low constant temperatures (0, 2.5, 5 and 7.5 °C) and five different high constant temperatures (32, 33, 34, 35 and 37 °C) either under a high (80–100%) or low (5–10%) relative humidity (RH). For each sampling duration, a set of 15 pupae randomly taken from the rearing stock was used. To produce high RH, a cotton ball saturated with water was placed at the bottom of a 50 mL closed centrifugation tube. For low RH, dehydrated silicagel was placed at the bottom of a 50 mL tube. Foam slices were added to the devices to prevent direct contact of SWD pupae with cotton or silicagel. RH and temperatures were checked directly into experimental tubes using Ibutton’s Hygrochron (Maxim Integrated, San Jose, CA, USA), and K thermocouples (measuring accuracy: ±0.5 °C) connected to Testo thermometers (Model 175 T3; TESTO Limited, Hampshire, England). As previously described, preliminary assays were first performed in order to calibrate the number and duration of sampling times to get emergence data spanning from 0 to 100%. Again, at least seven durations were used for each tested temperature (details in Table S5). Flies were considered as alive when the adult eclosed from the puparium.

### Statistical analyses

Survival data were modeled in R (R Core Team, 2016) by specifying a generalized linear model (GLM) with logistic link function for proportions outcome (i.e., number of dead/alive per vial). The response variable was dependent on stress duration, temperature, sex (for adults), RH treatment (when tested), and all the interactions. Full factorial models were used, and the effects of each variable were analyzed through an Analysis of Deviance (“Anova” function in “car” package, (Fox & Weisberg, 2011). The 50% median lethal times (Lt_50_) for each temperature were calculated as follow:

\begin{eqnarray*}{\mathrm{Lt}}_{50}= \frac{\mathrm{logit} \left( 0.5 \right) -a}{b} \quad \text{Venables {\XMLAMP} Ripley (2002)}. \end{eqnarray*}

where *a* and *b* respectively correspond to the intercept and the slope of each condition GLM’s prediction. 95% confidence intervals around estimated Lt_50_ were retrieved by resampling model parameters (10,000 iterations, “arm” package, Gelman & Su, 2014). Lt_50_ values represent the time at which 50% of the tested individuals are dead at a given temperatures. Even if is a standard and useful proxy to describe and compare thermal tolerance data, it is important to consider the entire range of probabilities and not only 50% survival. Therefore, to complement this information, the predicted values acquired from GLMs as function of both stress intensity and duration were also represented using 3D plots, following the thermal tolerance landscape (TTL) approach suggested by Rezende, Castañeda & Santos (2014). All the 3D representations of time × temperature × survival are available in Figs. S1 and S2. Finally, to help interpreting all the terms of the GLMs, effect plot function in the package “effects” (Fox, 2003) was used. These effect plots show the conditional coefficients (“marginal effects”) for all variables and interaction terms. All the effect plots are available in Figs. S3–S8 for each experiment separately.

## Results

### SWD cold tolerance

**Figure 1:**
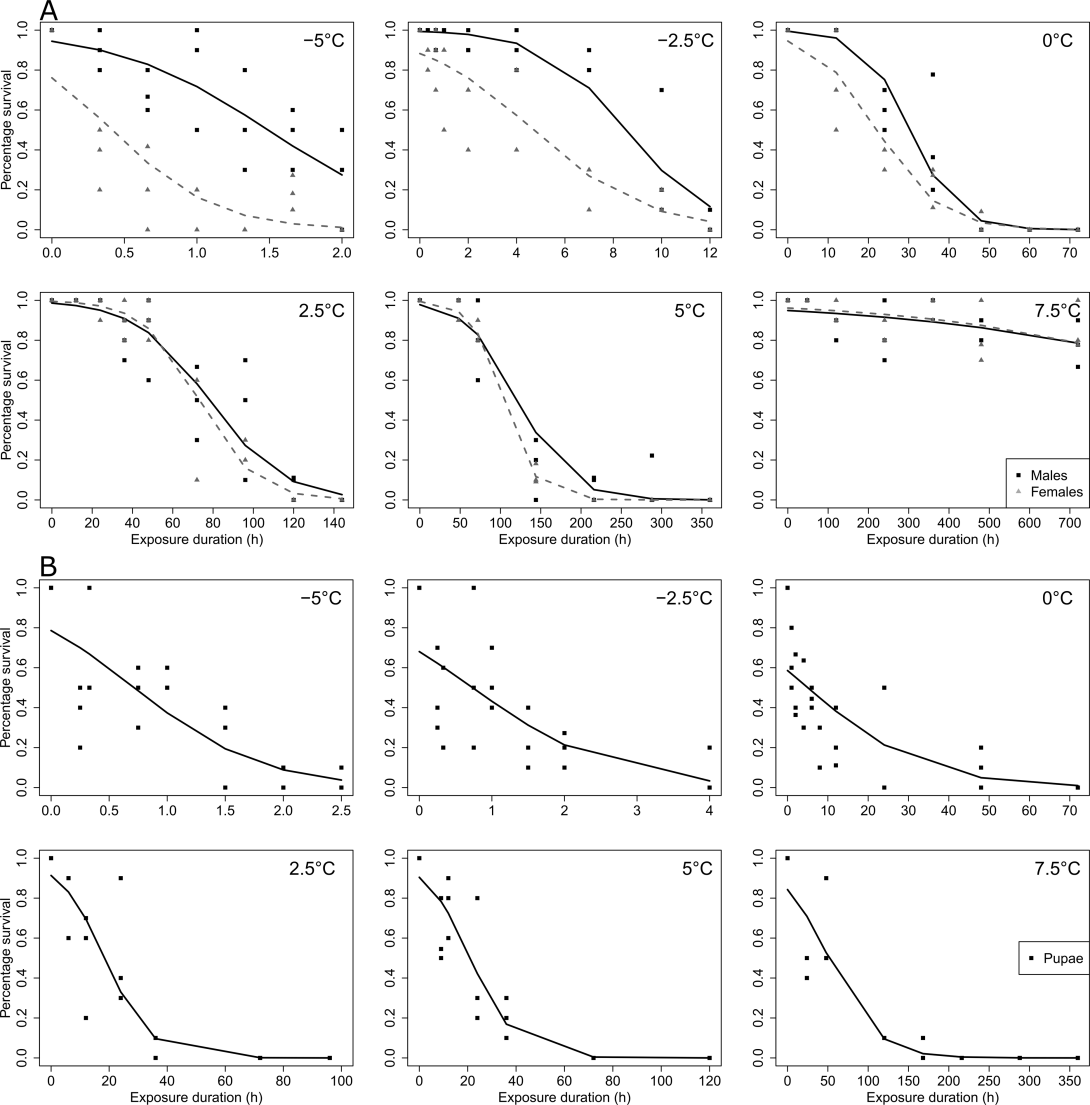
SWD survival as a function of low temperature and duration exposure. (A) adults; (B) pupae. Points correspond to observed data, and lines to GLM predictions. In (A), black solid lines correspond to male’s predictions, and grey dashed lines to females predictions. In (B) black solid lines correspond to pupae’s predictions. The tested temperatures are indicated in the left top corner of each plot.

Control mortality of untreated pupae didn’t exceed 1%. In both adults and pupae, 100% mortality was reached for all tested temperatures, except for adults kept at 7.5 °C. The multiple panels Fig. 1 illustrates cold survival data in adults (males and females, Fig. 1A) and in pupae (Fig. 1B) according to the different tested temperatures and durations. Temperature and duration had strong effects on adult cold survival (χ^2^ = 856.36, *df* = 5, *p* < 0.001; χ^2^ = 502.59, *df* = 1, *p* < 0.001, respectively). Survival decreased with decreasing temperature and with increasing exposure duration (Fig. 1A, Figs. S1 and S3). Furthermore, at the lowest temperatures, temporal reduction of survival was greatly reduced (duration × temperature interaction; χ^2^ = 1075.71, *df* = 5, *p* < 0.001; Figs. S1 and S3). Males were globally more cold-tolerant than females (χ^2^ = 99.95, *df* = 1, *p* < 0.001; Figs. S1 and S3). Sexes were differentially affected by decreasing temperatures (temperature × sex interaction; χ^2^ = 41.63, *df* = 5, *p* < 0.001), with females more affected than males at the lowest tested temperatures (Fig. 1A, Figs. S1 and S3). Temporal changes of survival were similar between sexes (no sex × duration interaction; χ^2^ = 0.65, *df* = 1, *p* > 0.05).

Temperature and duration also had a strong impact on pupal cold survival (χ^2^ = 156.68, *df* = 5, *p* < 0.001; χ^2^ = 463.94, *df* = 1, *p* < 0.001, respectively) (Fig. 1B). Survival significantly decreased with decreasing temperature and with increasing exposure duration (Fig. 1B, Figs. S1 and S4). The temporal decrease in survival was dependent on temperature (duration × temperature interaction; χ^2^ = 161.43, *df* = 5, *p* < 0.001), it was much faster at lower temperatures (Fig. 1B, Figs. S1 and S4).

**Figure 2:**
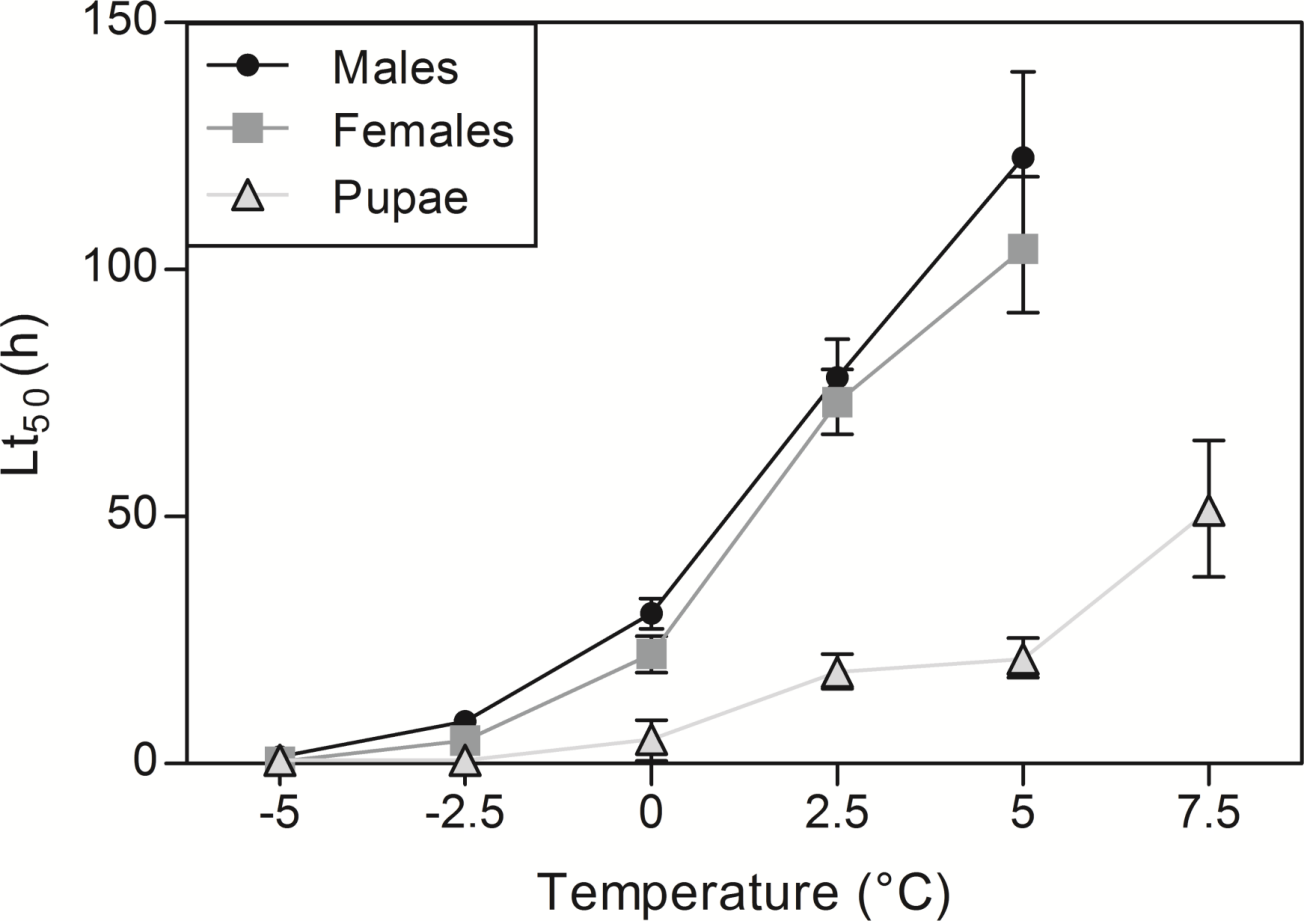
Estimated Lt_50_ values for each tested low temperature. Males, females and pupal Lt_50_ values ± 95% confidence intervals. Lt_50_ is the time at which 50% of the population is dead. Lt_50_ values are calculated from GLMs.

Lt_50_ for adults and pupae at the different tested low temperatures are provided in Fig.  2. Based on Lt_50_ values and their confidence intervals, pupae appeared much less cold-tolerant than adults (Fig. 2). For adults, models weren’t able to estimate Lt_50_ at 7.5 °C, as 80% of flies survived the 30 days of cold exposure (i.e., 50% mortality was not reached) (see Fig. 1A).

### SWD heat tolerance

**Figure 3:**
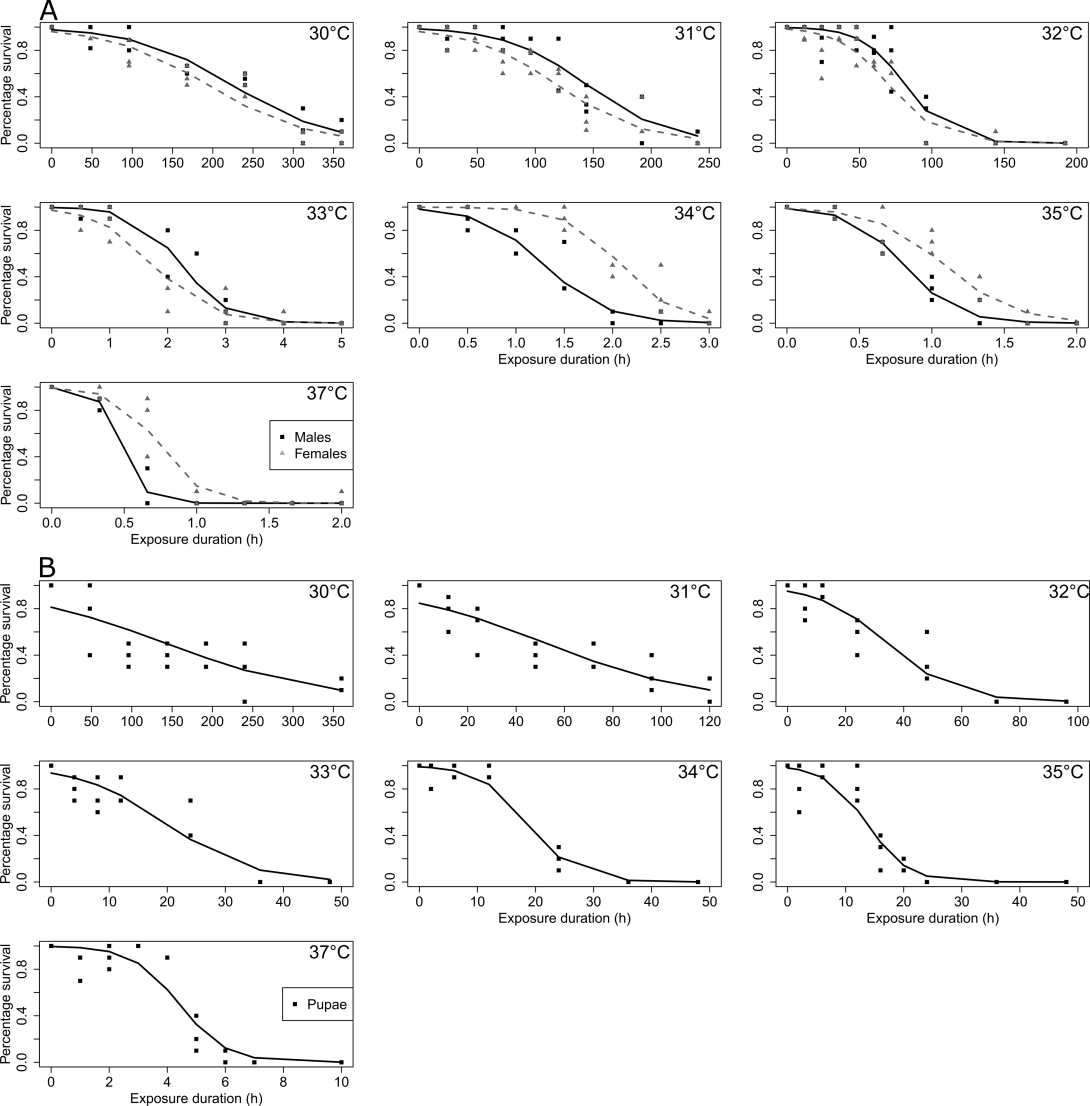
SWD survival as a function of high temperature and duration exposure. (A) adults; (B) pupae. Points correspond to observed data, and lines to GLM predictions. In (A), black solid lines correspond to male’s predictions, and grey dashed lines to female’s predictions. In (B), black solid lines correspond to pupae’s predictions. The tested temperatures are indicated in the left top corner of each plot.

For both adults and pupae, 100% mortality was reached for all tested temperatures, except for pupae at 30 and 31 °C, where five and four individuals, respectively, eclosed during the last day of the experiment and were then considered as alive. The multiple panels Fig. 3 illustrates survival data in adults (males and females, Fig. 3A) and in pupae (Fig. 3B) according to the different tested temperatures and durations. Temperature and duration had strong effect on adult heat survival (χ^2^ = 819.69, *df* = 6, *p* < 0.001; χ^2^ = 889.77, *df* = 1, *p* < 0.001, respectively). Survival decreased with increasing temperature and increasing exposure duration (Fig. 3A and Fig. S5 ). Furthermore, at highest temperatures, temporal reduction of survival was much faster than at milder temperatures (duration × temperature interaction; χ^2^ = 1495.20, *df* = 6, *p* < 0.001; Figs. S1 and S5). There was no difference between males and females (χ^2^ = 1.52, *df* = 1, *p* > 0.05). Yet sexes were differentially affected by increasing temperature (temperature × sex interaction; χ^2^ = 94.43, *df* = 6, *p* < 0.001), with males more affected than females at the highest temperatures (Fig. 3A, Figs. S1 and S5), indeed at 34, 35 and 37 °C, mid-time survival of female was at least twice greater than that of males (Fig. 3A). Temporal decreases of survival were similar between sexes (no sex × duration interaction, χ^2^ = 0.19, *df* = 1, *p* > 0.05).

Temperature and duration also had a strong impact on pupal heat survival (χ^2^ = 210.72, *df* = 6, *p* < 0.001; χ^2^ = 388.71, *df* = 1, *p* < 0.001, respectively) (Fig. 3B). Survival significantly decreased with increasing temperature and with increasing exposure duration (Fig. 3B, Figs. S1 and S6). The temporal decrease in survival was dependent on temperature (duration × temperature interaction; χ^2^ = 662.25, *df* = 6, *p* < 0.001), it was also much faster at higher tested temperatures. This latter effect is illustrated by the duration of tests which clearly reduced as the tested temperature increased (e.g., max 100 h at 32 °C *vs.* max 10 h at 37 °C) (Fig. 3B).

**Figure 4:**
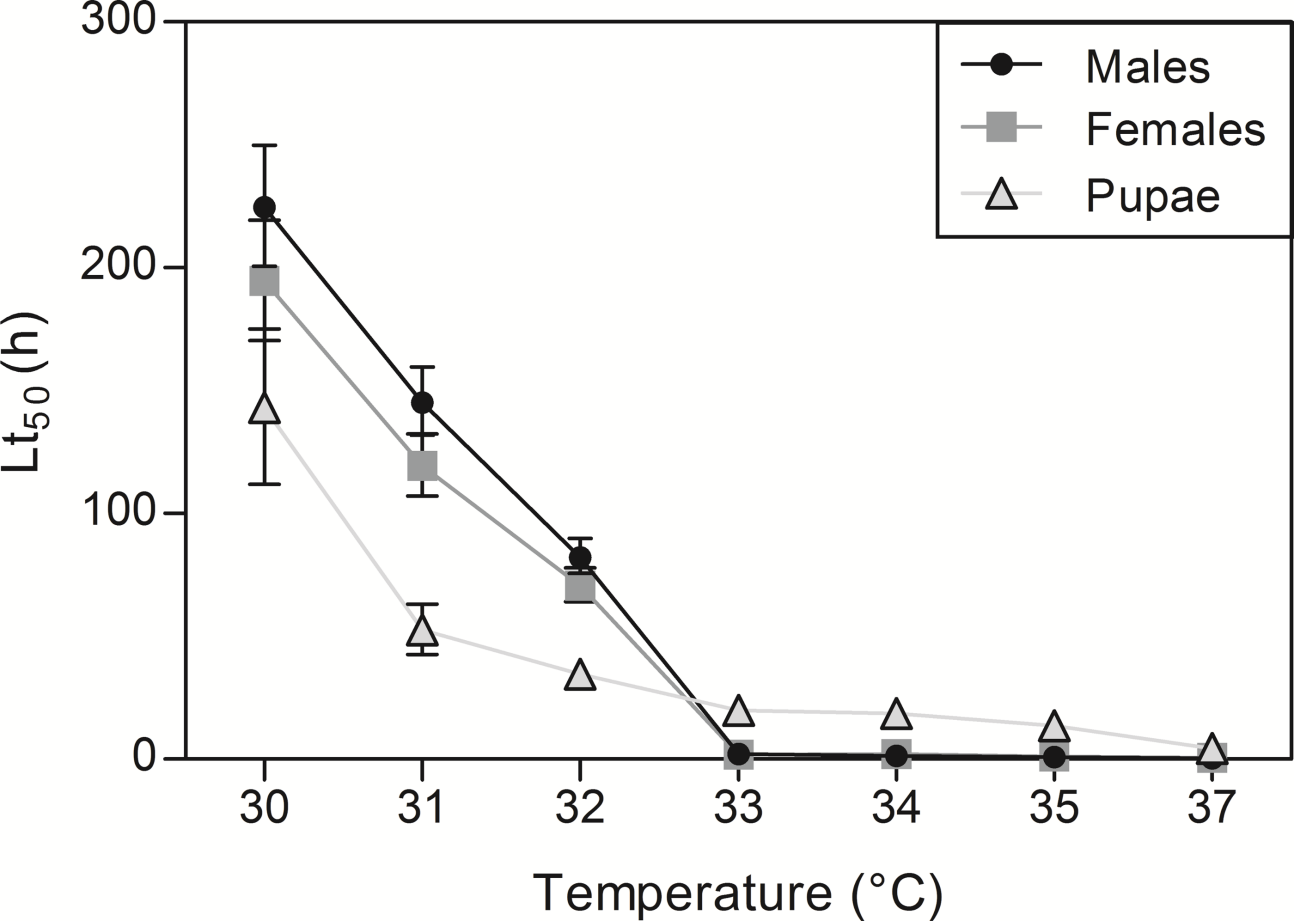
Estimated Lt_50_ values for each tested high temperature. Males, females and pupal Lt_50_ values ± 95% confidence intervals. Lt_50_ isthe time at which 50% of the population is dead. Lt_50_ values are calculated from GLMs.

Lt_50_ for adults and pupae at the different high temperatures are provided in Fig. 4. Based on Lt_50_ values and their confidence intervals, pupae appeared much less tolerant than adults at temperatures under 33 °C; however, they were more tolerant than adults at temperatures above 33 °C (Fig. 4).

### Interaction between relative humidity and thermal stress

**Figure 5:**
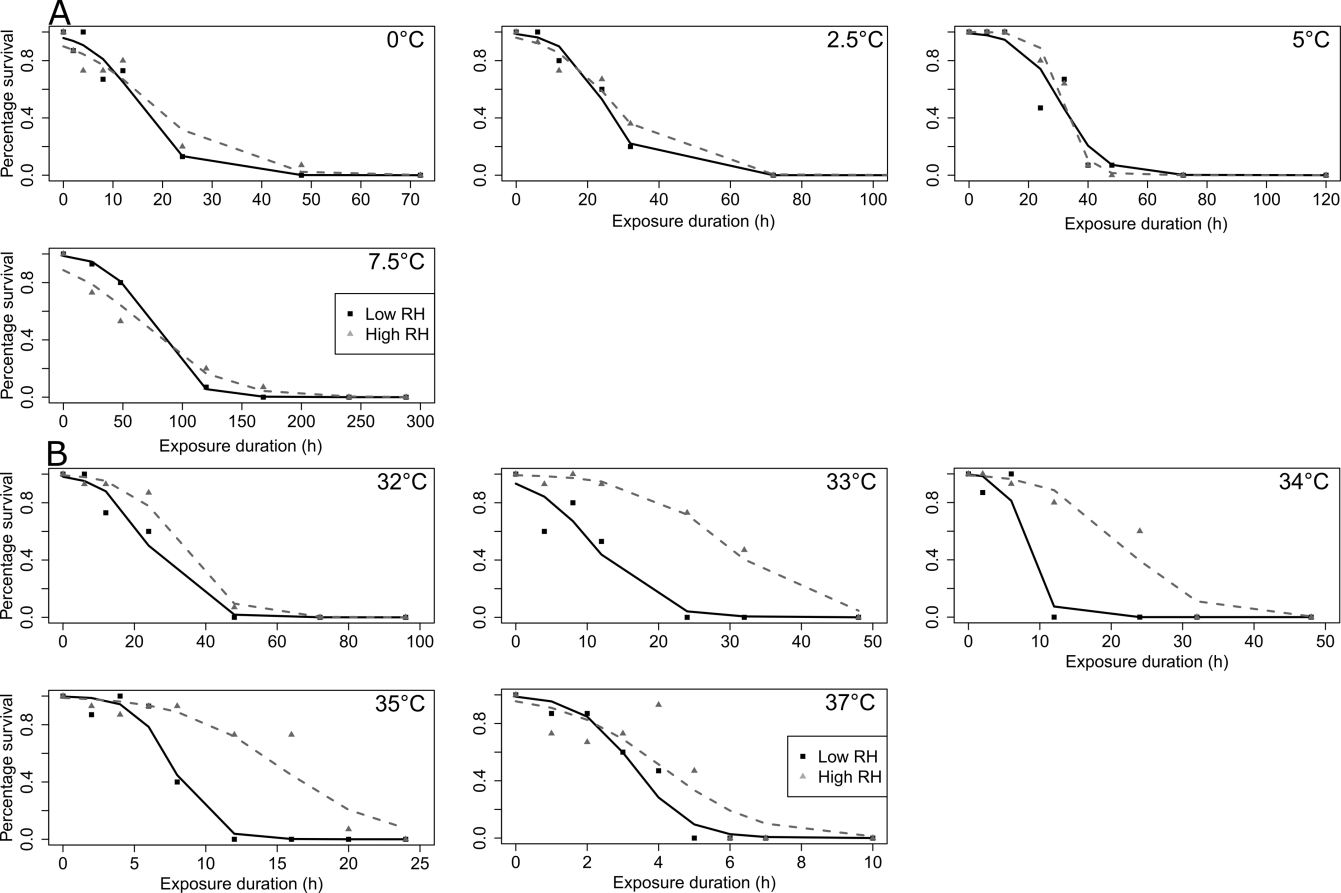
SWD pupal survival as a function of temperature and exposure duration under two relative humidity (RH) levels. (A) Cold exposure; (B) Heat exposure. Points correspond to observed data, and lines to GLM predictions. The tested temperatures are indicated in the left top corner of each plot. Black solid lines: low RH, grey dashed lines: high RH.

Low and high temperature treatments were statistically analyzed separately. Under cold exposure, 100% mortality was reached for all tested temperatures for both high and low RH. The multiple panels Fig. 5 illustrates survival data in pupae according to the different tested temperatures (A: cold; B: heat) and durations. Temperature and duration had strong effect on pupal cold survival (χ^2^ = 91.74, *df* = 3, *p* < 0.001; χ^2^ = 649.88, *df* = 1, *p* < 0.001, respectively). Cold survival decreased with decreasing temperature and with increasing exposure duration (Fig. 5A, Figs. S2 and S7). Furthermore, at the lowest temperatures, temporal reduction of survival was much faster (duration ×  temperature interaction; χ^2^ = 68.07, *df* = 3, *p* < 0.001; Figs. S2 and S7). RH did not differentially affect cold survival (χ^2^ = 0.02, *df* = 1, *p* > 0.05), but temporal changes were different between RH levels: survival decrease was slightly faster in dry than in humid condition (RH × duration interaction; χ^2^ = 5.48, *df* = 1, *p* < 0.05; Figs. S2 and S7).

**Figure 6:**
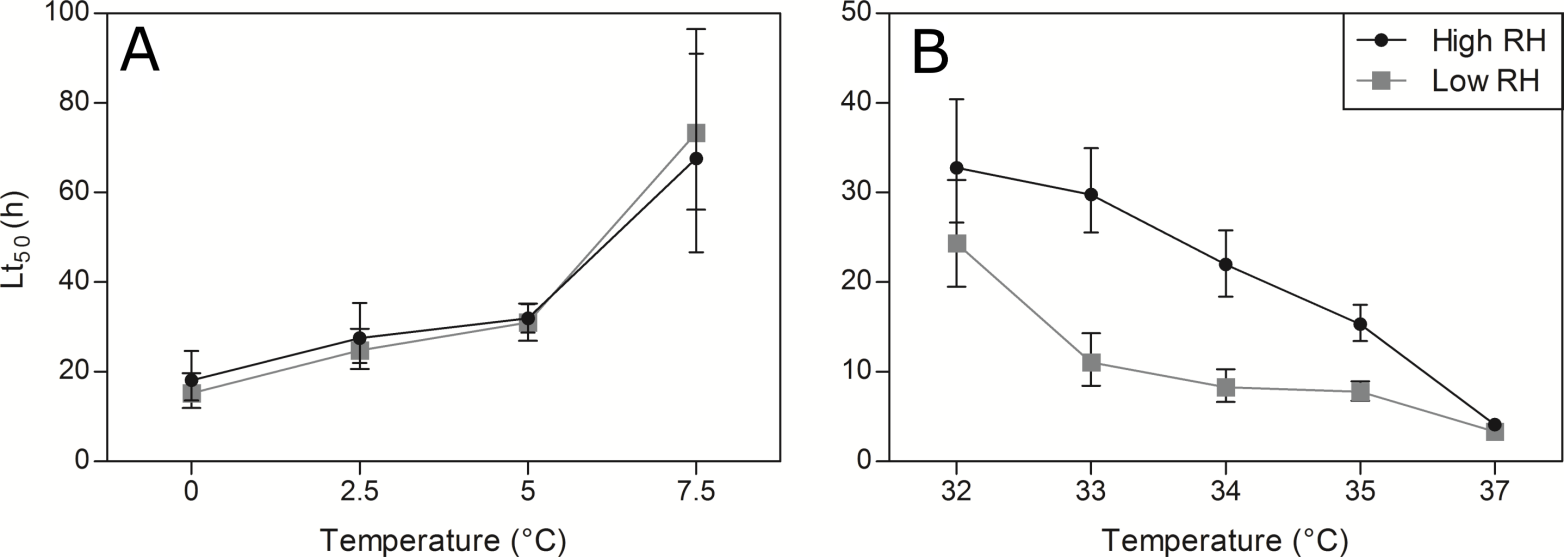
Pupal Lt_50_ values at low and high temperatures under two relative humidity (RH) levels. Lt_50_ values ± 95% confidence interval values for low (A) and high temperatures (B). Lt_50_values are calculated from previous GLM predictions.

Under heat exposure, 100% mortality was also reached for all tested temperatures for both high and low RH (Fig. 5B). Temperature and duration had again strong effects on pupae heat survival (χ^2^ = 306.20, *df* = 4, *p* < 0.001; χ^2^ = 831.90, *df* = 1, *p* < 0.001, respectively). Survival decreased with increasing temperature and with increasing exposure duration (Fig. 5B, Figs. S2 and S8). Furthermore, at the highest temperatures, temporal reduction of survival was much faster (duration × temperature interaction; χ^2^ = 83.46, *df* = 4, *p* < 0.001). RH greatly affected heat survival (χ^2^ = 95.97, *df* = 1, *p* < 0.001), with survival being significantly higher when pupae were exposed to high *vs*. low RH (Fig. 5B, Figs. S2 and S8). In addition, RH interacted with both temperature and duration (χ^2^ = 19.25, *df* = 4, *p* < 0.001; χ^2^ = 14.17, *df* = 1, *p* < 0.001, respectively). Survival decreased with increasing temperature and this thermal-dependent process was more severe under low RH (Fig. 5B, Figs. S2 and S8). In addition, temporal decrease in survival (across all temperatures) was globally faster under low RH. Based on Lt_50_ values and their confidence intervals, it appeared that low RH greatly diminished heat survival, but did not affect cold survival (Fig. 6).

## Discussion

In the present study, the basal thermal tolerance of SWD was studied considering adult and pupal survival as a function of both stress intensity (heat and cold) and exposure duration. A reduction in survival with increasing thermal stress intensity and duration was observed, both under low and high temperatures. This decrease is consistent with the classical dose–response relationship where survival declines with the “dose of stress” which is considered here as a combination of temperature stress intensity and duration (Colinet, Lalouette & Renault, 2011; Rezende, Castañeda & Santos, 2014).

The cold tolerance of SWD estimated in the present study was rather consistent with previously reported data. Indeed, it appeared that adult and pupal mortality occurred very rapidly at subzero temperatures (see Figs. 1 and 2). This fits with early reports that also found very short survival durations at subzero temperatures (*e.i.* Jakobs, Gariepy & Sinclair, 2015; Stephens et al., 2015; Plantamp et al., 2016; Ryan et al., 2016). These data corroborate that SWD is a chill susceptible species that does not tolerate brief exposures to sub-zero temperatures (Kimura, 2004; Dalton et al., 2011; Jakobs, Gariepy & Sinclair, 2015; Ryan et al., 2016; Plantamp et al., 2016). At 0 °C, it required about one day to reach 50% mortality in adults and much less time was needed (approximatively 5 h) in pupae. This is also in the range of previous data on SWD (Kimura, 2004; Jakobs, Gariepy & Sinclair, 2015; Plantamp et al., 2016); however, the values described in this study appear slightly lower than those reported in other studies and strains. These slight variations in thermotolerance may be related to different rearing conditions. Indeed, we reared flies at 25 °C not at 21 °C as in other laboratories (Jakobs, Gariepy & Sinclair, 2015; Plantamp et al., 2016). Variations may also result from different local adaptations of the tested strains (Hoffmann, Anderson & Hallas, 2002; Van Heerwaarden et al., 2012). Finally, our strain was reared under laboratory conditions since 2011, and hence, we cannot exclude an effect of inbreeding on stress tolerance, despite constant efforts to mitigate this issue. At temperatures above 0 °C, several days were required before reaching Lt_50_. Interestingly, there appeared to be a sort of threshold between 5 and 7.5 °C where individuals shifted from detrimental condition (at 5 °C) to non-injurious condition (at 7.5 °C). Indeed, at 5 °C, Lt_50_ was reached in only five days, while at 7.5 °C, mortality remained low (under 20%) even after rather long exposure (one month). Previous data reported that non-acclimated SWD adults start to fall into coma at temperatures just below 5 °C (Jakobs, 2014). Therefore, it is likely that this temperature represents a physiological limit under which chilling injuries, such as neuromuscular dysfunctions, start to accumulate (Hazell & Bale, 2011; MacMillan et al., 2012). In temperate regions, cold snaps with freezing events could be lethal to SWD. However, it seems that SWD overwinters as adults by migrating into protected microclimates, in leaf litter or in human made structures (Kanzawa, 1939; Kimura, 2004; Dalton et al., 2011; Zerulla et al., 2015; Rossi-Stacconi et al., 2016). This avoiding strategy likely allows SWD to escape low winter temperatures *in natura*, and colonize new cool regions (Rota-Stabelli, Blaxter & Anfora, 2013; Asplen et al., 2015).

Because thermal performance curves are nonlinear and asymmetric (skewed towards low temperatures) (Martin & Huey, 2008; Colinet et al., 2015a), we predicted uneven effects of increasing the intensity of cold *vs.* heat stress. Essentially, we assumed that SWD survival will decrease rather progressively with increasing cold stress intensity, and we expected a steep decline in survival over certain limits under heat stress. Observation of Lt_50_ (Figs. 2 and 4) values and TTLs patterns (Fig. S1) support this assumption. Under low temperature conditions, a progressive survival decline was observed (Fig. 2 and Fig. S1), while under high temperature stress, there was clearly a limit over which survival crashed suddenly and became close to zero (Fig. 4 and Fig. S1). Indeed, at 32 °C, adult flies could sustain continuous heat stress for several days (Lt_50_ of 3–4 days), whereas at 33 °C, most flies succumbed within a couple of hours (Lt_50_ of about 2 h). Therefore 32 °C seem to be very close to critical thermal maximum for survival of SWD. Characteristically, the drop in performance (i.e., survival in this case) is generally more precipitous at supra-optimal temperatures than at sub-optimal temperatures (Denlinger & Yocum, 1998). Arrhenius-like effects can explain progressive and reversible changes of performance at sub-optimal temperatures, while the sharp decline at supra-optimal temperatures is generally ascribed to the destabilizing effects of heat on molecular interactions such as irreversible protein denaturation (Schulte, Healy & Fangue, 2011). These results are in accordance with early studies performed on different SWD populations. Kanzawa (1939) noticed a motor activity decrease of SWD when exposed to 30 °C, and Kimura (2004) estimated that the 50% lethal temperature at heat was around 32°C. Also, the upper thermal limit for development is estimated to be at 31.5 °C (Asplen et al., 2015). Ryan et al. (2016) found no adult hatching when Canadian flies developed at 31 °C. Therefore, our survival data together with the previous literature suggest that SWD is not a particularly heat-tolerant species. This likely explains the very low field survival of SWD when temperatures exceed 30 °C (Dalton et al., 2011; Tochen et al., 2014). While overwintering strategy is rather well studied (Kanzawa, 1936; Stephens et al., 2015; Shearer et al., 2016; Toxopeus et al., 2016; Wallingford & Loeb, 2016), how flies cope with heat stress in the fields and more generally how SWD flies manage to survive under summer conditions (i.e., heat coupled with desiccation) is currently unknown.

Differences in thermotolerance between sexes and stages were expected. Under low temperature, SWD males were slightly more cold-tolerant than females. This contrasts with previous SWD studies which reported that females were more cold-tolerant than males (Kimura, 2004; Dalton et al., 2011; Jakobs, Gariepy & Sinclair, 2015). However, lack of difference in cold tolerance between sexes were also reported in SWD (Ryan et al., 2016). In *D. melanogaster*, the sex effect on cold tolerance can be sometimes in favor of males (Kelty & Lee, 2001; Sejerkilde, Sørensen & Loeschcke, 2003; Jensen, Overgaard & Sørensen, 2007), or females (David et al., 1998; Condon et al., 2015). These discrepancies may result from various factors, such as different measures of cold tolerance, tested temperatures or age of flies (Jensen, Overgaard & Sørensen, 2007). In consequence, our results suggest that in SWD, as in *D. melanogaster*, sexual dimorphism in various metrics of cold tolerance appears more as an idiosyncratic than a general rule (Gibert & Huey, 2001). This view is also supported by our observations that, under high temperature, we detected an interaction (sex × temperature) that suggested that females better tolerated heat exposure but at certain temperatures (i.e., at the greatest temperatures).

Based on previous data from *D. melanogaster* (Jensen, Overgaard & Sørensen, 2007), we predicted that pupae would be less cold-tolerant than adults. Indeed, it appeared that pupal Lt_50_ values under cold conditions were consistently much lower than values of adults at all tested temperatures; and this was clearly visible on the shape of TTLs (Fig. S1). Furthermore, exposure to 7.5 °C induced high pupal mortality while it hardly affected adults. Other studies suggest that SWD pupae are less cold-tolerant than adults. For instance, Dalton et al. (2011) reported that pupae died earlier than adults when exposed to temperatures below 10 °C, and Ryan et al. (2016) found that SWD pupae could not survive 42 d of cold exposure contrary to adults. This seems coherent with the observation that in SWD, the overwintering stage is the adult in reproductive diapause (Stephens et al., 2015; Zerulla et al., 2015; Shearer et al., 2016; Toxopeus et al., 2016; Rossi-Stacconi et al., 2016; Wallingford & Loeb, 2016). Ontogenetic differences under high temperature revealed different patterns than under low temperature. Pupae appeared less tolerant than adults to prolonged mild heat stress (temperatures < 33 °C), while under severe heat stress conditions (temperatures > 33 °C), pupae could sustain heat stress for much longer than adults. Indeed, under acute heat stress, pupae managed to survive exposures for several hours (from 10 to 20 h depending on the temperature), while adults succumbed in less than 2 h. In *Drosophila buzzatii*, pupae seem to be the most heat resistant stage, surviving temperatures above those that would kill all the other life stages (Krebs & Loeschcke, 1995). The high heat tolerance of pupae might be explained by Bogert effect (Huey, Hertz & Sinervo, 2003). According to this principle, behavioral thermoregulation allows ectothermic animals to escape lethal temperatures, thus less mobile stages (like pupae) have to cope with and tolerate more extremes conditions than mobile stages (Marais & Chown, 2008; Mitchell, Sinclair & Terblanche, 2013).

Finally, we predicted that contrasted RH levels will affect thermal tolerance patterns. Specifically, we expected that highly desiccating conditions during thermal stress will further reduce survival compared to stress conditions under high RH. As predicted, RH had strong impact on pupal survival, but this manifested only under heat stress. The Lt_50_ values illustrate these marked differences (Figs. 6A and 6B). Under cold conditions, the shapes of TTLs were globally identical between low and high RH, whereas under heat conditions, the TTLs were extended towards longer survival under high RH (Fig. S2). In *D. melanogaster*, the humidity during both heat (Bubliy et al., 2012) and cold exposure (Kobey & Montooth, 2013) alters survival rate. Combining two stressors like high temperature with low RH provides more stressful conditions to vinegar flies than high temperature with high RH (Bubliy et al., 2012). Here, the same synergetic-like phenomena was observed. Prince & Parsons (1977) showed that under low RH, *D. melanogaster* adults move towards lower temperatures likely to mitigate water loss. It is reasonable to speculate that in natural conditions, SWD adults also tend to avoid heat stress and low RH by searching for protected and favorable microhabitats, but this remains to be tested. Recent mark-capture researches indicate that SWD achieves short-distance migrations from field margins to cultivated crops (Klick et al., 2016); therefore, migration towards favorable microclimates is completely conceivable. During the pupal stage, however, flies are immobile and are thus potentially subjected to prolonged heat and desiccation stress, particularly if pupation occurs outside of infested fruits, as is the case with SWD (Asplen et al., 2015). Despite being protected within the puparium, water loss by pupae can strongly affect survival of drosophilids, and even moderately dry conditions can induce substantial pupal mortality at permissive temperature (Kojima & Kimura, 2003). A recent study from Tochen et al. (2016) indicates that low RH (e.g., 20%) induced poor survival and lack of reproduction in SWD, suggesting that this species is particularly sensitive to water loss. Under low temperature, there was globally no effect of RH on pupal cold survival. Death during prolonged cold exposure may be due to a combination of stressors: low temperature, starvation and desiccation. If pupae were suffering from desiccation at cold, then altering RH during low temperature exposure should affect water loss, and therefore, the survival duration at cold. Lack of RH effect at cold suggests that desiccation is not a primary cause of mortality under cold stress.

In this work, basal thermal tolerance of SWD was studied considering survival as a function of temperature stress intensity (under heat and cold) and exposure duration in adults (males and females) and in pupae. It appeared that survival under heat and cold conditions was dependent on both stress intensity and duration, and hence, this study provides a comprehensive description and visualization of SWD thermal tolerance and limits. These results confirmed that SWD is a chill susceptible species. We found that at temperatures over 5 °C, adults managed to survive for rather long periods (one month). Tolerance to thermal stress over a range of conditions showed rather different perspectives: a sudden *vs.* a more progressive survival decline under heat *vs.* cold conditions, respectively. In particular, 32 °C seemed to be very close to critical thermal maximum for survival of SWD. A sexual dimorphism in thermal tolerance was also found but was temperature-dependent. Difference in thermal tolerance were also observed between stages, with pupae being drastically more sensitive to cold stress but more resistant to extreme heat stress than adults. Finally, data suggested that level of RH had strong impact on pupal survival under heat stress but not under cold stress.

Recently, a consortium of scientists has published a useful review with the updated situation of SWD all over the world (Asplen et al., 2015). The authors suggested a few directions for future research to improve the accuracy of SWD management. Acquisition of novel data on the biology of SWD at low temperature was highlighted as a priority, and we believe the present dataset may provide valuable elements in this regard. The present study is one of the first to provide a global description of SWD basal thermal tolerance, especially bringing new information about heat stress tolerance and the interaction between temperature and relative humidity. In a context of global climate change and considering the winter temperature warming on temperate region, these results together with previous data from the literature highlight that SWD will most likely be able to tolerate mild cold stress conditions for several consecutive days (e.g., one month to 7.5 °C). This capacity may contribute to its overwintering success in novel invaded temperate regions. However, we wish to draw attention to the fact that stress tolerance data acquired from field-collected populations may deeply contrast with those resulting from laboratory-adapted lines (*e.i.* Hoffmann et al., 2001; Schou, Loeschcke & Kristensen, 2015). In consequence, despite logistical constraints, the next needed step is the realization of thermal studies on field-collected individuals.

##  Supplemental Information

10.7717/peerj.3112/supp-1 Figure S1 Thermal tolerance landscapes for low and high temperatures Males (A), females (B) and pupal (C) cold tolerance landscapes, and Males (D), females (E) and pupal (F) heat tolerance landscapes. Points are observed values, and surfaces correspond to GLMs predictions (Binomial GLM, link = logit).

10.7717/peerj.3112/supp-2 Figure S2 Thermal tolerance landscapes for low and high temperatures under two relative humidity (RH) levels Cold tolerance landscapes under low (A) and high (B) relative humidity, and heat tolerance landscapes under low (C) and high (D) relative humidity. Points corresponds to observed values, and surfaces corresponds to GLMs predictions (Binomial GLM, link = logit).

10.7717/peerj.3112/supp-3 Figure S3 Effect plots from GLM on the experiment with adults exposed to low temperatures The plots show the conditional coefficients (“marginal effects”) of all variables included in the model as well as effects resulting from the interaction terms. The variables are cold exposure temperature, duration, sex (Male vs Female) and all the interactions. The statistical outputs (from the table of deviance) are also given (in blue) before the plots for all terms of the model.

10.7717/peerj.3112/supp-4 Figure S4 Effect plots from GLM on the experiment with pupae exposed to low temperatures The plots show the conditional coefficients (“marginal effects”) of all variables included in the model as well as effects resulting from the interaction terms. The variables are cold exposure temperature, duration, and all the interactions. The statistical outputs (from the table of deviance) are also given (in blue) before the plots for all terms of the model.

10.7717/peerj.3112/supp-5 Figure S5 Effect plots from GLM on the experiment with adults exposed to high temperatures The plots show the conditional coefficients (“marginal effects”) of all variables included in the model as well as effects resulting from the interaction terms. The variables are heat exposure temperature, duration, sex (Male vs Female) and all the interactions. The statistical outputs (from the table of deviance) are also given (in blue) before the plots for all terms of the model.

10.7717/peerj.3112/supp-6 Figure S6 Effect plots from GLM on the experiment with pupae exposed to high temperatures The plots show the conditional coefficients (“marginal effects”) of all variables included in the model as well as effects resulting from the interaction terms. The variables are heat exposure temperature, duration and all the interactions. The statistical outputs (from the table of deviance) are also given (in blue) before the plots for all terms of the model.

10.7717/peerj.3112/supp-7 Figure S7 Effect plots from GLM on the experiment with pupae exposed to low temperatures under two relative humidity levels The plots show the conditional coefficients (“marginal effects”) of all variables included in the model as well as effects resulting from the interaction terms. The variables are cold exposure temperature, duration, RH and all the interactions. The statistical outputs (from the table of deviance) are also given (in blue) before the plots for all terms of the model.

10.7717/peerj.3112/supp-8 Figure S8 Effect plots from GLM on the experiment with pupae exposed to high temperatures under two relative humidity levels The plots show the conditional coefficients (“marginal effects”) of all variables included in the model as well as effects resulting from the interaction terms. The variables are heat exposure temperature, duration, RH and all the interactions. The statistical outputs (from the table of deviance) are also given (in blue) before the plots for all terms of the model.

10.7717/peerj.3112/supp-9 Table S1 Temperatures and respective exposure durations used for adult’s cold tolerance assays

10.7717/peerj.3112/supp-10 Table S2 Temperatures and respective exposure durations used for pupae’s cold tolerance assays

10.7717/peerj.3112/supp-11 Table S3 Temperatures and respective exposure durations used for adult’s heat tolerance assays

10.7717/peerj.3112/supp-12 Table S4 Temperatures and respective exposure durations used for pupae’s heat tolerance assays

10.7717/peerj.3112/supp-13 Table S5 Temperatures and respective exposure durations used for pupae’s thermal stress assays under high or low relative humidity

## Additional Information and Declarations

### Data Availability

The following information was supplied regarding data availability: Data: https://figshare.com/s/e94eacc9d64dbbd47941. R scripts: https://figshare.com/s/dba3a9c7eecb1d4603f3.
