# Supplementary figures and images for: Basal tolerance to heat and cold exposure of the spotted wing drosophila, Drosophila suzukii
Source: PeerJ. 2017 Mar 23;5:e3112. doi: 10.7717/peerj.3112 (PMC5366067; doi:10.7717/peerj.3112)

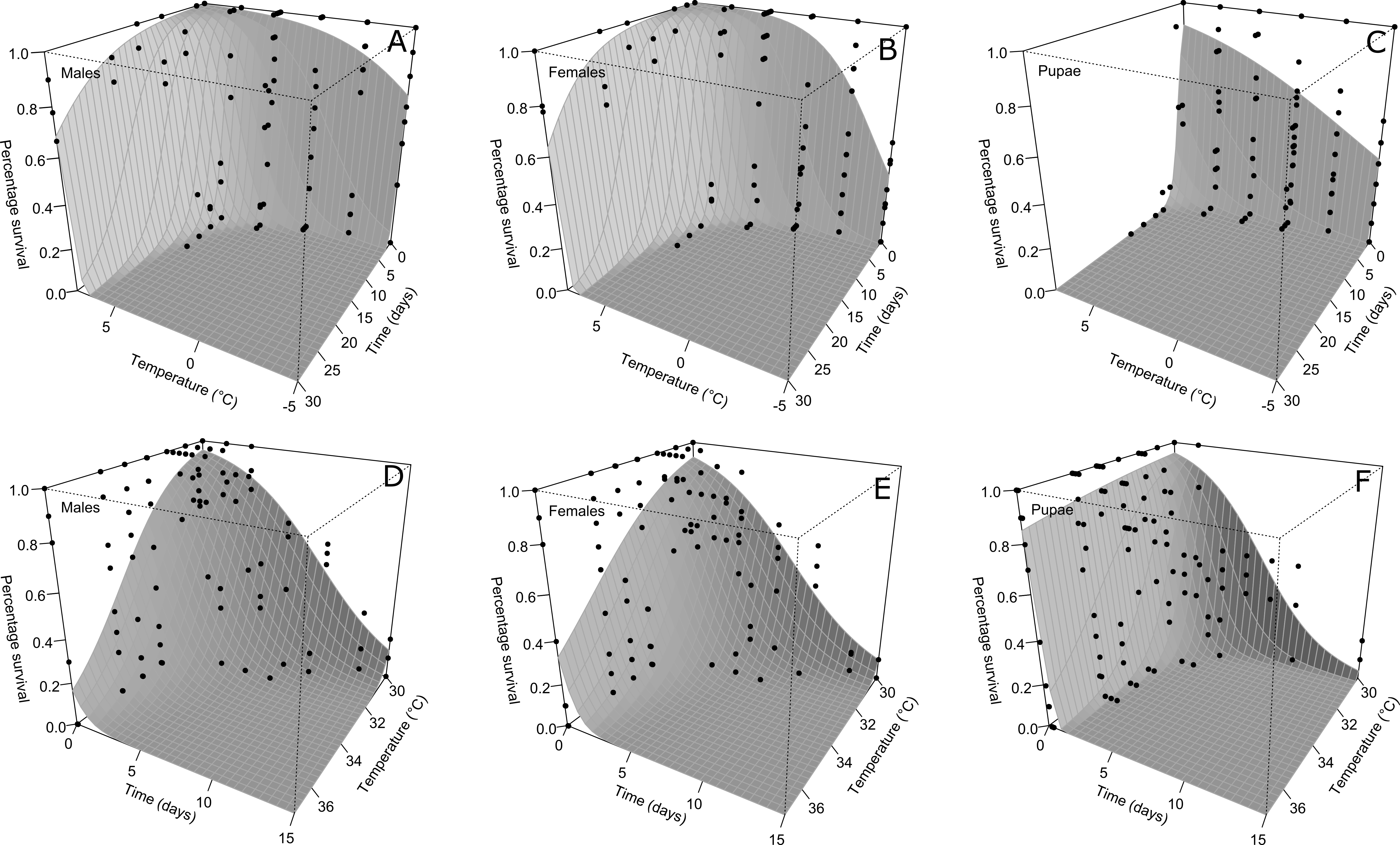

Supplement: Figure S1 — Males (A), females (B) and pupal (C) cold tolerance landscapes, and Males (D), females (E) and pupal (F) heat tolerance landscapes. Points are observed values, and surfaces correspond to GLMs predictions (Binomial GLM, link = logit). [file peerj-05-3112-s001.png]

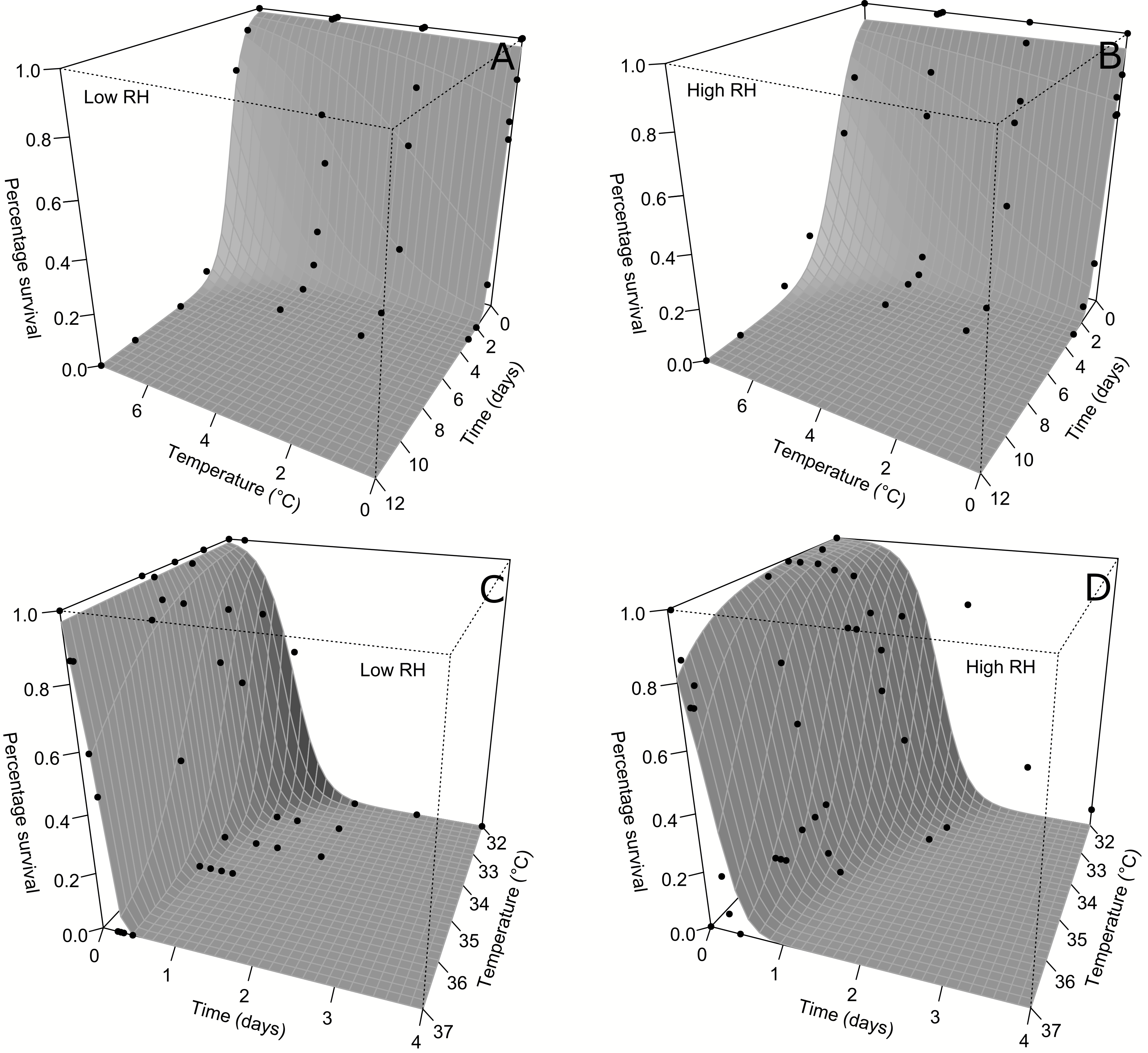

Supplement: Figure S2 — Cold tolerance landscapes under low (A) and high (B) relative humidity, and heat tolerance landscapes under low (C) and high (D) relative humidity. Points corresponds to observed values, and surfaces corresponds to GLMs predictions (Binomial GLM, link = logit). [file peerj-05-3112-s002.png]

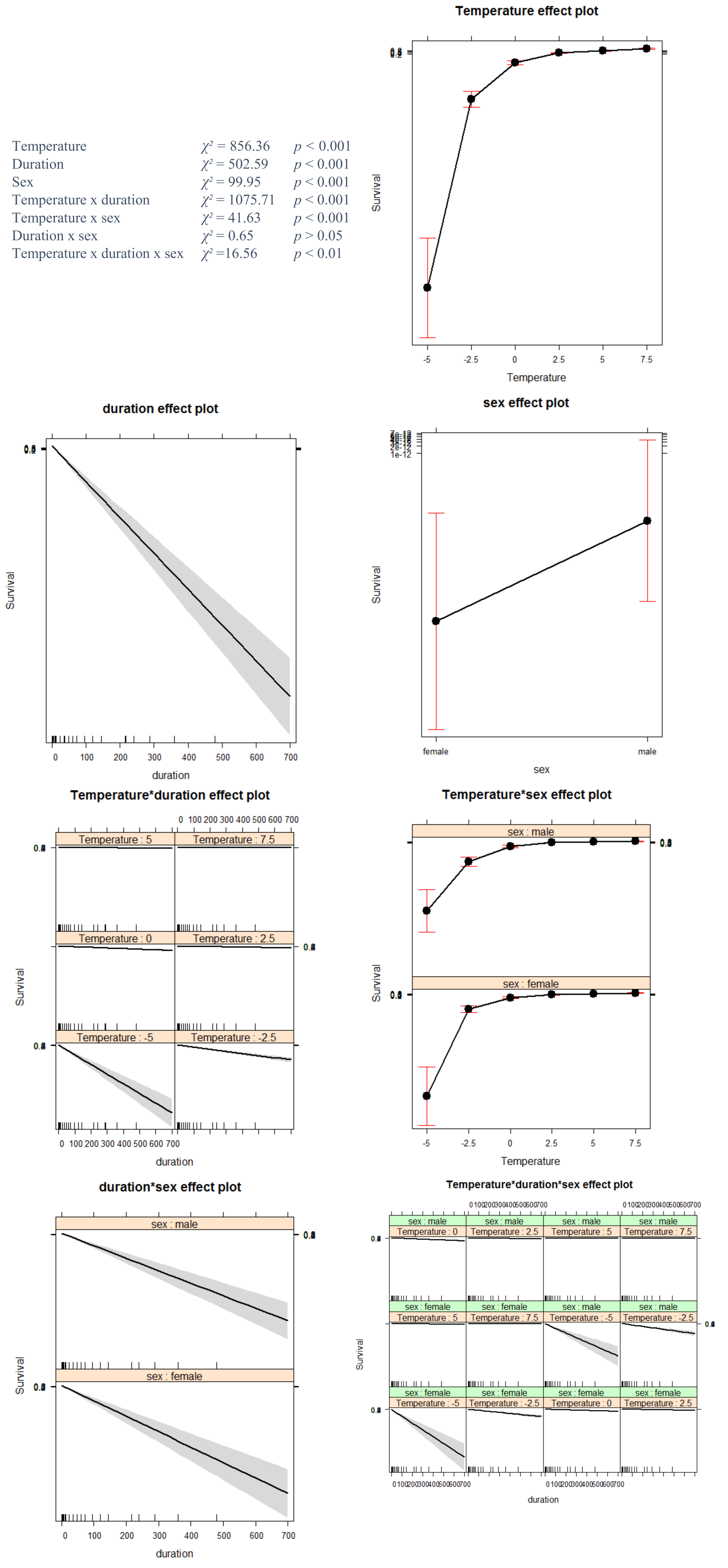

Supplement: Figure S3 — The plots show the conditional coefficients (“marginal effects”) of all variables included in the model as well as effects resulting from the interaction terms. The variables are cold exposure temperature, duration, sex (Male vs Female) and all the interactions. The statistical outputs (from the table of deviance) are also given (in blue) before the plots for all terms of the model. [file peerj-05-3112-s003.png]

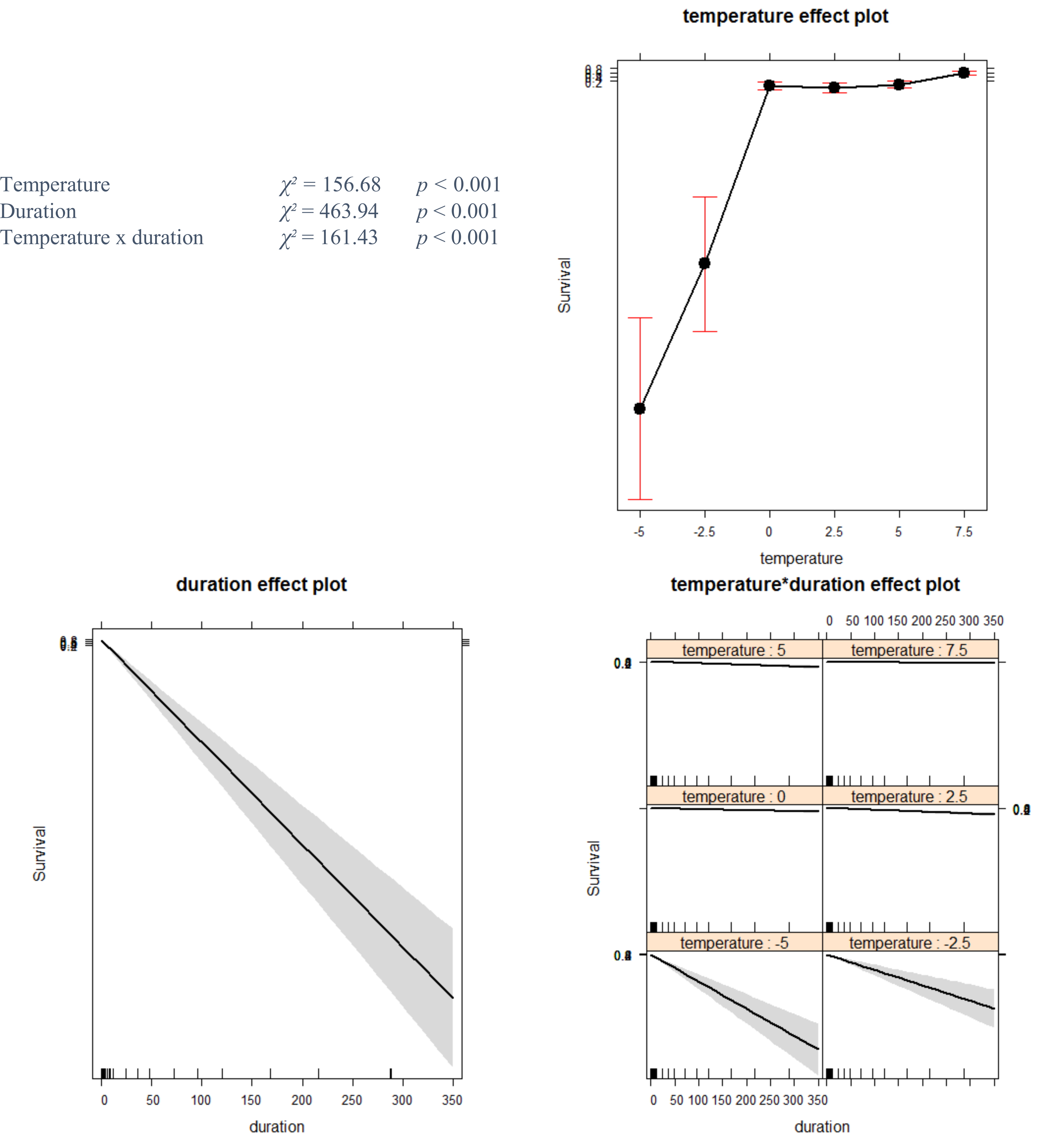

Supplement: Figure S4 — The plots show the conditional coefficients (“marginal effects”) of all variables included in the model as well as effects resulting from the interaction terms. The variables are cold exposure temperature, duration, and all the interactions. The statistical outputs (from the table of deviance) are also given (in blue) before the plots for all terms of the model. [file peerj-05-3112-s004.png]

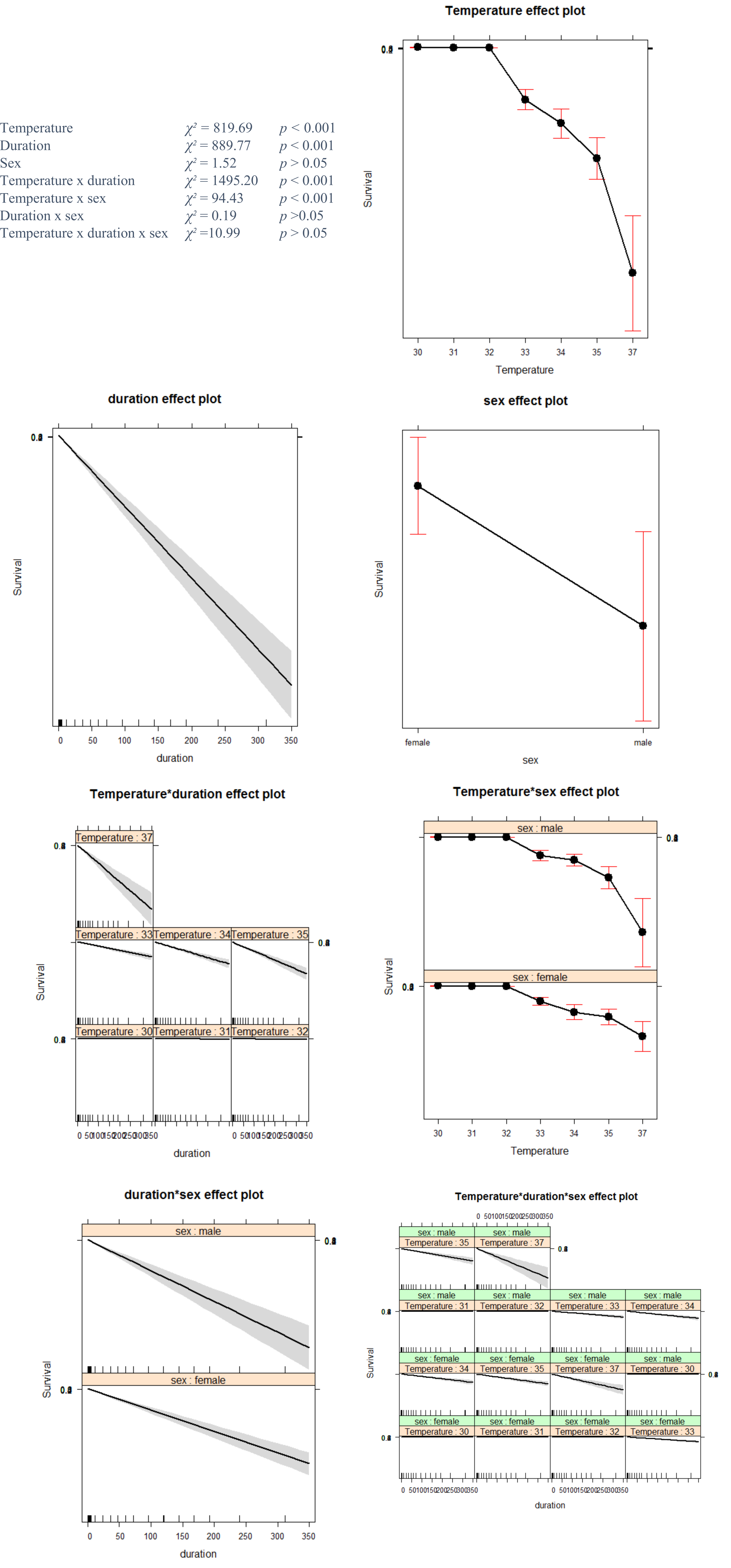

Supplement: Figure S5 — The plots show the conditional coefficients (“marginal effects”) of all variables included in the model as well as effects resulting from the interaction terms. The variables are heat exposure temperature, duration, sex (Male vs Female) and all the interactions. The statistical outputs (from the table of deviance) are also given (in blue) before the plots for all terms of the model. [file peerj-05-3112-s005.png]

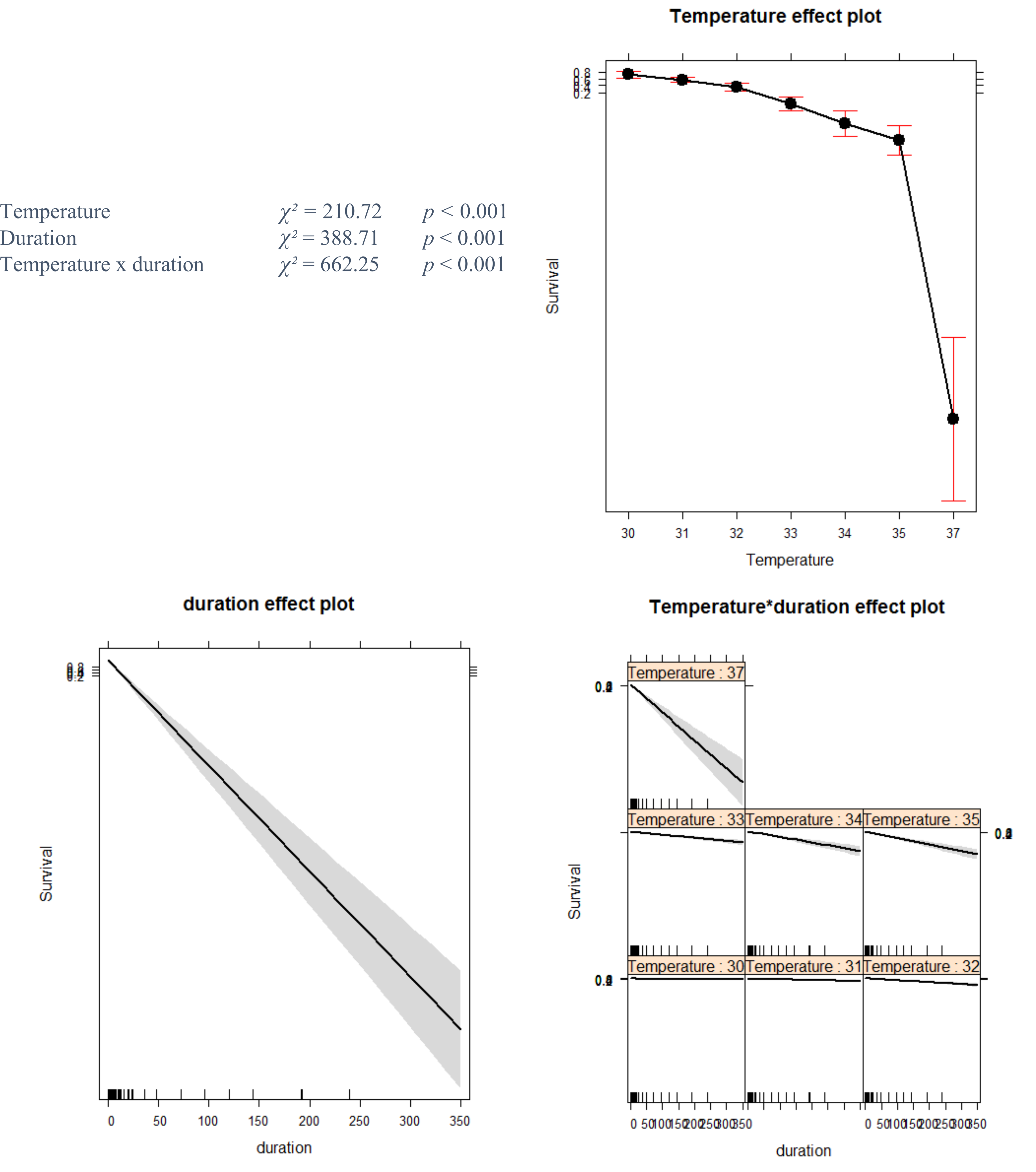

Supplement: Figure S6 — The plots show the conditional coefficients (“marginal effects”) of all variables included in the model as well as effects resulting from the interaction terms. The variables are heat exposure temperature, duration and all the interactions. The statistical outputs (from the table of deviance) are also given (in blue) before the plots for all terms of the model. [file peerj-05-3112-s006.png]

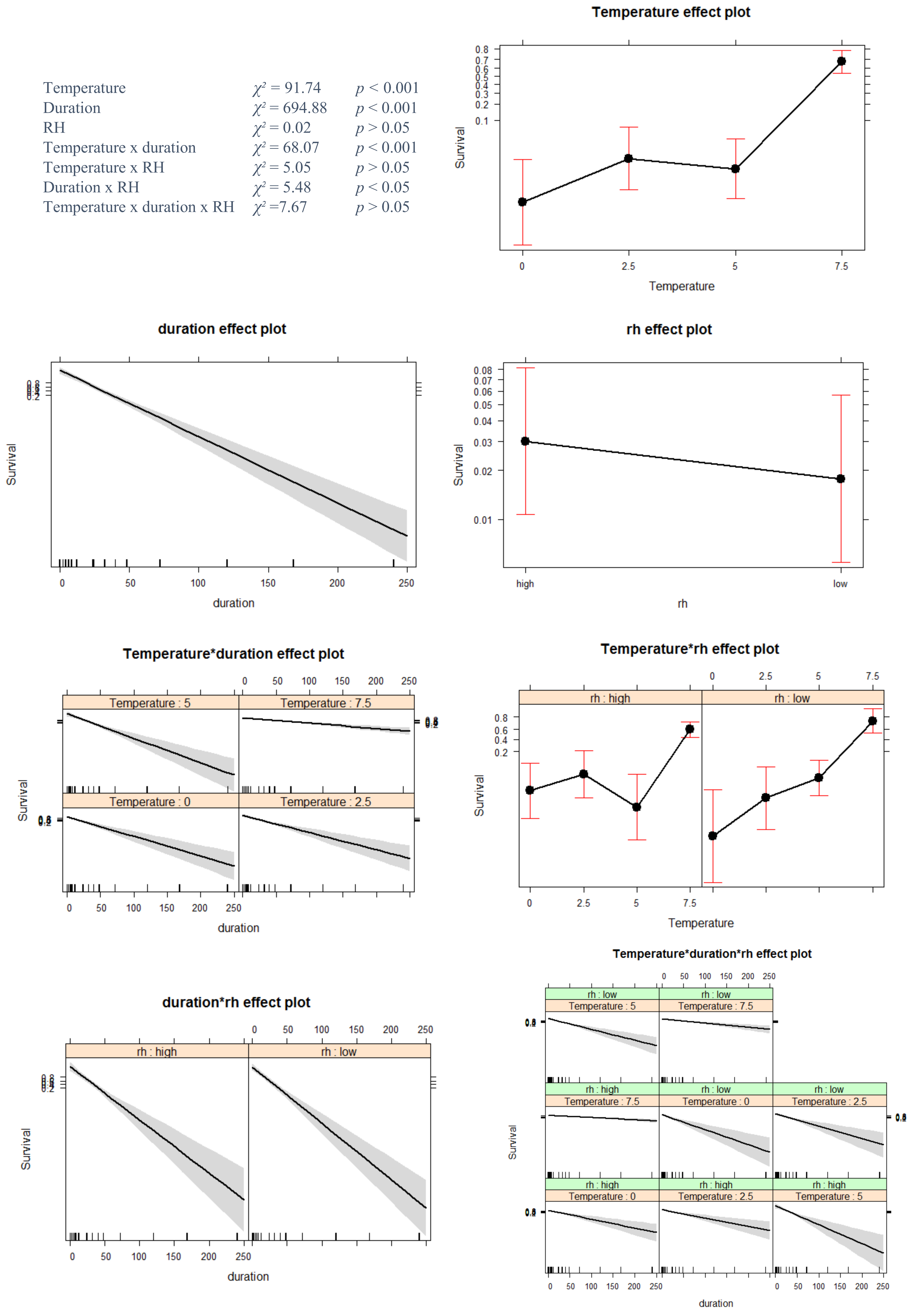

Supplement: Figure S7 — The plots show the conditional coefficients (“marginal effects”) of all variables included in the model as well as effects resulting from the interaction terms. The variables are cold exposure temperature, duration, RH and all the interactions. The statistical outputs (from the table of deviance) are also given (in blue) before the plots for all terms of the model. [file peerj-05-3112-s007.png]

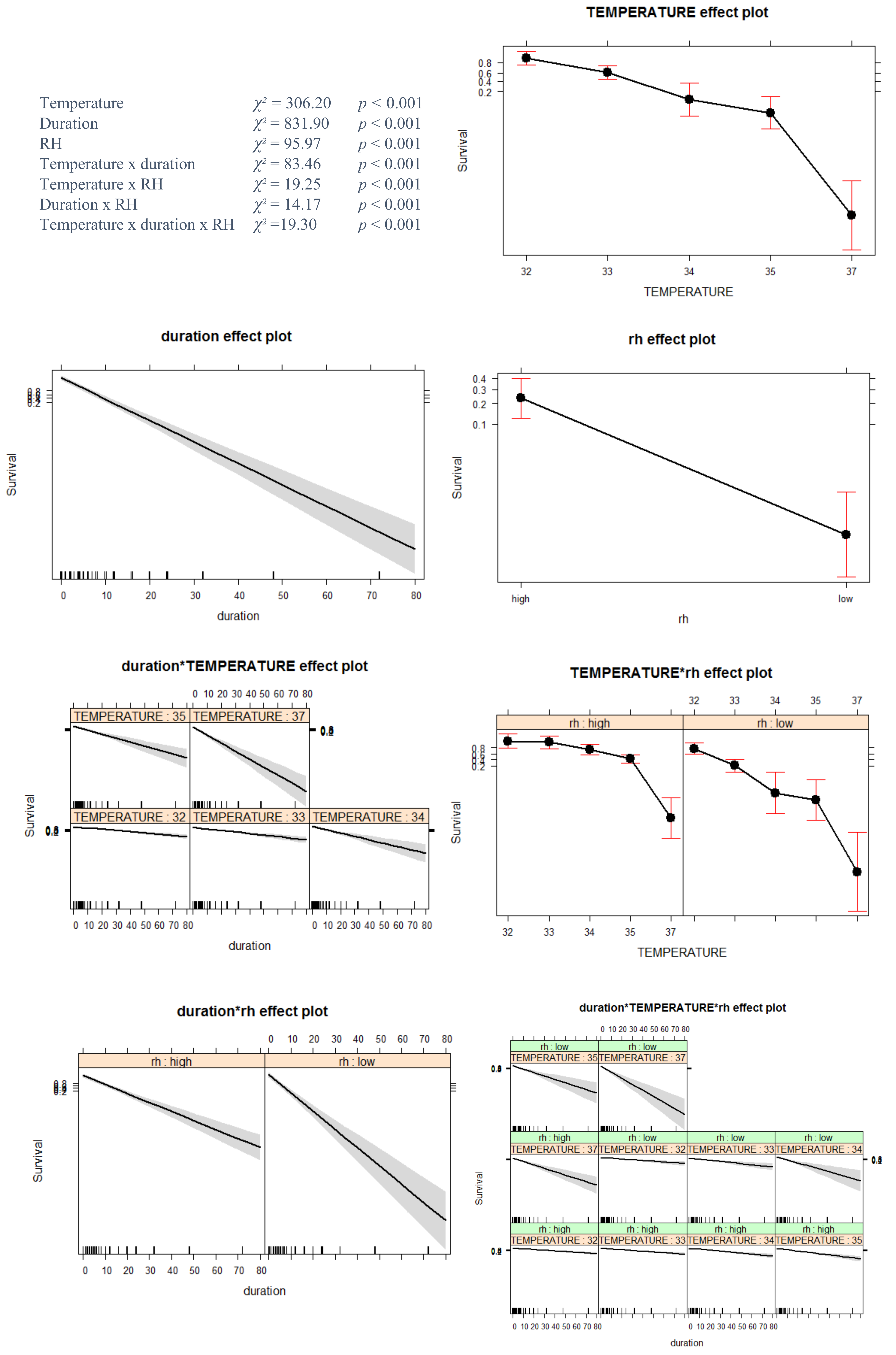

Supplement: Figure S8 — The plots show the conditional coefficients (“marginal effects”) of all variables included in the model as well as effects resulting from the interaction terms. The variables are heat exposure temperature, duration, RH and all the interactions. The statistical outputs (from the table of deviance) are also given (in blue) before the plots for all terms of the model. [file peerj-05-3112-s008.png]
