# Supplementary material for: Basal tolerance to heat and cold exposure of the spotted wing drosophila, Drosophila suzukii
Source: PeerJ. 2017 Mar 23;5:e3112. doi: 10.7717/peerj.3112 (PMC5366067; doi:10.7717/peerj.3112)
Supplement: Table S1 [file peerj-05-3112-s009.pdf]

Temperatures and respective exposure durations used for adult's cold tolerance assays.

-5 °C	-2.5 °C	0 °C	2.5 °C	5 °C	7.5 °C
20 min	20 min	12 h	12 h	2 d	2 d
40 min	40 min	24 h	24 h	3 d	5 d
60 min	1 h	36 h	36 h	6 d	10 d
80 min	2 h30	48 h	48 h	9 d	15 d
100 min	4 h	60 h	3 d	12 d	20 d
2 h	7 h	3 d	4 d	15 d	30 d
	10 h				
	12 h				
