# Supplementary material for: Basal tolerance to heat and cold exposure of the spotted wing drosophila, Drosophila suzukii
Source: PeerJ. 2017 Mar 23;5:e3112. doi: 10.7717/peerj.3112 (PMC5366067; doi:10.7717/peerj.3112)
Supplement: Table S2 [file peerj-05-3112-s010.pdf]

Temperatures and respective exposure durations used for pupae's cold tolerance assays.

-5 °C	-2.5 °C	0 °C	2.5 °C	5 °C	7.5 °C
15 min	15 min	2 h	6 h	6 h	1 d
30 min	30 min	4 h	12 h	12 h	2 d
45 min	45 min	8 h	24 h	24 h	5 d
1 h	1 h	12 h	36 h	36 h	7 d
1 h30	1 h30	24 h	3 d	3 d	10 d
2 h	2 h	48 h	4 d	5 d	12 d
2 h30	4 h	72 h			
