# Supplementary material for: Basal tolerance to heat and cold exposure of the spotted wing drosophila, Drosophila suzukii
Source: PeerJ. 2017 Mar 23;5:e3112. doi: 10.7717/peerj.3112 (PMC5366067; doi:10.7717/peerj.3112)
Supplement: Table S3 [file peerj-05-3112-s011.pdf]

Temperatures and respective exposure durations used for adult's heat tolerance assays.

30 °C	31 °C	32 °C	33 °C	34 °C	35 °C	37 °C
2 d	1 d	12 h	30 min	30 min	20 min	20 min
4 d	2 d	24 h	1 h	1 h	40 min	40 min
7 d	3 d	36 h	2 h	1 h30	60 min	60 min
10 d	4 d	48 h	3 h	2 h	80 min	80 min
13 d	5 d	60 h	4 h	2 h30	100 min	100 min
15 d	6 d	3 d	5 h	3 h	2 h	2 h
	8 d	4 d				
	10 d	6 d				
		8 d				
