# Supplementary material for: Basal tolerance to heat and cold exposure of the spotted wing drosophila, Drosophila suzukii
Source: PeerJ. 2017 Mar 23;5:e3112. doi: 10.7717/peerj.3112 (PMC5366067; doi:10.7717/peerj.3112)
Supplement: Table S4 [file peerj-05-3112-s012.pdf]

Temperatures and respective exposure durations used for pupae's heat tolerance assays.

30 °C	31 °C	32 °C	33 °C	34 °C	35 °C	37 °C
2 d	12 h	6 h	4 h	2 h	2 h	1 h
4 d	24 h	12 h	8 h	6 h	6 h	2 h
6 d	2 d	24 h	12 h	12 h	12 h	3 h
8 d	3 d	48 h	24 h	24 h	24 h	4 h
10 d	4 d	60 h	36 h	36 h	36 h	5 h
15 d	5 d	4 d	48 h	48 h	48 h	6 h
						7 h
						10 h
